# Proper modelling of ligand binding requires an ensemble of bound and unbound states

**DOI:** 10.1107/S2059798317003412

**Published:** 2017-03-06

**Authors:** Nicholas M. Pearce, Tobias Krojer, Frank von Delft

**Affiliations:** a Structural Genomics Consortium, University of Oxford, Roosevelt Drive, Oxford OX3 7DQ, England; b Diamond Light Source Ltd, Harwell Science and Innovation Campus, Didcot OX11 0QX, England; c Department of Biochemistry, University of Johannesburg, Aukland Park, Johannesburg 2006, South Africa

**Keywords:** crystallography, modelling, ligand binding, model validation

## Abstract

Although noncovalent binding by small molecules cannot be assumed *a priori* to be stoichiometric in the crystal lattice, occupancy refinement of ligands is often avoided by convention. Occupancies tend to be set to unity, requiring the occupancy error to be modelled by the *B* factors, and residual weak density around the ligand is necessarily attributed to ‘disorder’. Where occupancy refinement *is* performed, the complementary, superposed unbound state is rarely modelled. Here, it is shown that superior accuracy is achieved by modelling the ligand as partially occupied and superposed on a ligand-free ‘ground-state’ model. Explicit incorporation of this model of the crystal, obtained from a reference data set, allows constrained occupancy refinement with minimal fear of overfitting. Better representation of the crystal also leads to more meaningful refined atomic parameters such as the *B* factor, allowing more insight into dynamics in the crystal. An outline of an approach for algorithmically generating ensemble models of crystals is presented, assuming that data sets representing the ground state are available. The applicability of various electron-density metrics to the validation of the resulting models is assessed, and it is concluded that ensemble models consistently score better than the corresponding single-state models. Furthermore, it appears that ignoring the superposed ground state becomes the dominant source of model error, locally, once the overall model is accurate enough; modelling the local ground state properly is then more meaningful than correcting all remaining model errors globally, especially for low-occupancy ligands. Implications for the simultaneous refinement of *B* factors and occupancies, and for future evaluation of the limits of the approach, in particular its behaviour at lower data resolution, are discussed.

## Abstract 2

The importance of modelling the superpositions of ligand-bound and unbound states that commonly occur in crystallographic data sets is emphasized and demonstrated. The generation of an ensemble that models not only the state of interest is important for the high-quality refinement of low-occupancy ligands, as well as to explain the observed density more completely.

## 1. Introduction   

Crystallographic diffraction experiments reveal the atomic composition of protein crystals, but when the crystal is composed of objects in multiple states the resulting diffraction pattern is a weighted average of these states. Ligands will often bind at sub-unitary occupancy in the crystal, as shown by examples where extensive experimental optimization is required to obtain interpretable electron density (McNae *et al.*, 2005 ▸; Müller, 2017 ▸): not only ligand affinity but also solubility and the crystal lattice play a part in determining the occupancy of a ligand (Danley, 2006 ▸). Noncovalent ligands are always subject to binding equilibrium, so in general, even crystal forms that form only by the co-crystallization of ligand and protein cannot be assumed to have the ligand bound at full occupancy, as even high-affinity ligands may be partially displaced by unpredictable experimental artefacts.

In crystals where a state of interest (usually bound ligand) remains at sub-unitary occupancy, the total electron density in that region is an average of this *bound state* and the complementary state, which we term the *ground state*. This latter state refers to those molecules in the lattice in which the ligand (or change) of interest is not present, but where all other crystal conditions are the same (the same pH, same molecular species present in the crystal *etc.*). Where the ground state of the crystal contains conformational heterogeneity, this state may be composed of multiple substates; these can still be collectively referred to as *the ground state*. (We avoid the term ‘unbound state’, as this state may still bind buffer, solvent or other molecules.)

**Figure 1:**
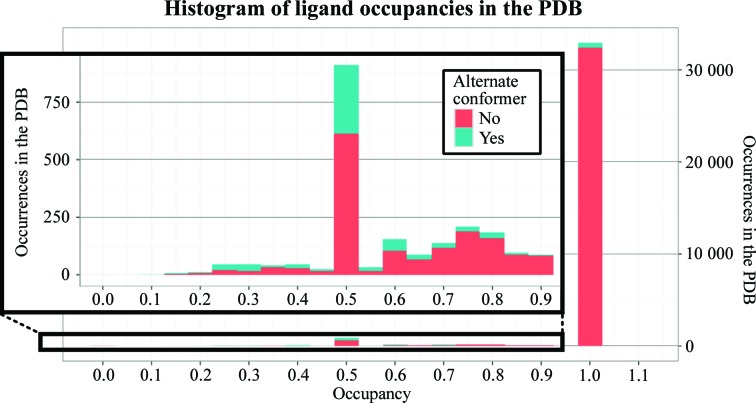
Most ligands in the PDB are modelled at unitary occupancy, and many partial occupancy ligands are not modelled with an alternate state. A histogram is shown of all ligand occupancies in the PDB classified by the presence of an alternate-conformer identifier (red, no conformer ID; blue, modelled with a conformer ID). Sub-unitary occupancy ligands are shown in the inset graph. Only the first instance of each ligand type from each PDB structure was used; following this all ligands with fewer than five non-H atoms or more than 50 instances were removed to avoid bias towards common molecules. Where alternate conformations of ligands are present, the total occupancy is used. The large majority of ligands are modelled in a single conformation at unitary occupancy (32 396, 92.1%). A smaller number have non-unitary occupancies but no alternate conformer identifier (1640, 4.7%). The remainder are modelled using alternate conformers (1122, 3.2%), of which 548 are ligands with alternate conformers that sum to unitary occupancy. Worryingly, there are also ten instances with more than 100% occupancy. These modelling statistics are unlikely to represent the true situation in crystal structures; ligands will always have a superposed solvent model when present at partial occupancy. The relationship between data-set resolution and the occupancy of partial occupancy ligands is shown in Supplementary Fig. S1; there is no clear trend towards occupancy refinement at high occupancy.

Nevertheless, it is standard practice in macromolecular crystallography not to model this superposition explicitly. An analysis of the PDB (Berman *et al.*, 2000 ▸, 2003 ▸) demonstrates that in the vast majority of cases only the ligand-bound conformation of the crystal is modelled, and generally at unitary occupancy (Fig. 1 ▸). In these cases, any occupancy error is absorbed by inflation of the refined *B* factors: multiple conversations in online discussion fora such as ResearchGate (2014 ▸, 2016 ▸) indicate that the main application of occupancy refinement is to reduce difference density when it appears over a ligand model.

There are certainly good practical reasons for fixing occupancies to unity: at even moderately high resolution, allowing occupancies to change is usually an unjustifiable over-parameterization, analogous to the selection of an inappropriately complex *B*-factor model (Merritt, 2012 ▸). The interdependencies, instabilities and ambiguities that arise from the simultaneous refinement of both *B* factors and occupancies are well understood and notorious, as these numbers are highly correlated and are numerically indistinguishable at moderate-to-low resolutions (Bhat, 1989 ▸). Methods for parameterizing and performing robust occupancy refinement remain an ongoing area of research.

Conversely, there is frequently strong evidence in the maps that unitary occupancy is unjustified. Unfortunately, it is generally overlooked that simply reducing the occupancies of the bound state effectively models the complementary fraction of the crystal by vacuum, which is clearly unjustified. The failure to include a superposed ground state in the crystallo­graphic model in regions of interest should therefore be considered to be a glaring omission.

We propose that the standard strategy for modelling sub-unitary occupancy ligands should be to include a superposed ground-state model in refinement, provided that the resolution is sufficiently high and an atomic ground-state model can be independently derived from a ground-state crystal. In this work, we show that for straightforward models it is simple to include such an explicit model of the ground state and that this yields both better models of the bound state and good evidence for the presence of the ground state. We show this for good resolutions (1.5–2 Å) and for a range of ligand occupancies (0.26–0.84), and in each case rely on a persuasive, independent model of the ground state being available.

## 2. Methods   

### 2.1. Generation and refinement of the ensemble model   

We generated ensemble models by directly transferring the relevant atoms from a ground-state model, finalized against a ground-state data set, to models of subsequent data sets of the same crystal form. Specifically, the ground-state model around the binding site of interest was combined with the ligand-bound model through the appropriate use of alternate conformers (discussed further in §[Sec sec2.1.1]2.1.1). Whilst identifying and interpreting a partially occupied bound state of a crystal is not easy in general, we used *PanDDA* (Pearce *et al.*, 2016 ▸), which addresses this problem by explicitly separating the superposition of states (further described in §[Sec sec2.1.2]2.1.2).

Refinement of the ensemble model was performed with the two states constrained into separate occupancy groups. After each cycle of full-model refinement, the different states of the ensemble model were modelled and visually validated in *Coot* (Emsley *et al.*, 2010 ▸). The ground and bound states were each modified only with reference to the original ground-state maps and *PanDDA* maps, respectively, and the validity of the complete model was assessed by refinement against the ligand-bound data set.

During the modelling and refinement process, the ground-state model of the crystal was considered to be a Bayesian prior, such that the ground-state structure is assumed not to change from crystal to crystal. This applies even if the ground state is not clearly discernible in the electron density; minor states will be ‘masked’ by superposed major states (as in §[Sec sec3.4]3.4), but they will still remain except where the ligand is truly unitary occupancy. The ground state should therefore only be removed from the crystallographic ensemble model if it causes problems in refinement (see §[Sec sec4.1]4.1).

#### 2.1.1. Systematic labelling of crystal states to maximize interpretability   

Locally heterogeneous crystals are modelled through the use of alternate conformers, which ascribe each modelled atom to a particular state of the crystal (also described in Pearce *et al.*, 2016 ▸). Only for globally heterogeneous atomic models are alternate *model* identifiers used (see, for example, Burnley *et al.*, 2012 ▸).

To simplify the generation of occupancy-refinement keywords, all atoms of the same state (bound or ground) are given the same conformer ID (or *altloc* or *altid*). Their occupancies can then be grouped during refinement, and the occupancies of the complementary states constrained to sum to unity. The advantage of such a modelling protocol is that it allows each state to be easily extracted simply by selecting a particular conformer from the ensemble. This is crucial to enable the use of the structure by noncrystallographers, to whom only the bound state is of interest: for their purposes, the superposed ground state is an experimental artefact caused by non-unitary ligand occupancy in the crystal.

For conformer labelling, we have used a convention that we believe will allow the least ambiguous identification of the multi-conformer model, namely by distinguishing bound and ground states with conformer ID labels that are not used elsewhere in the structure. This aims to prevent the potential association of similarly labelled alternate conformers that are causally unrelated (for example side-chain flips on a distant surface site). Thus, in the case of a single conformer each for the bound state and the ground state, but where alternate conformers elsewhere in the structure and un­elated to binding are labelled *A* and *B* only, all ground-state-only atoms are set to conformer *C* and all bound-state-only atoms are set to conformer *D*. This labelling is only applied to atoms in the region affected by binding.

This protocol works well where there is one conformer in each of the bound and ground states, but a generalized method remains the subject of ongoing work to ensure that all cases (involving multiple conformers in either state) result in valid models (see §[Sec sec4.1]4.1).

#### 2.1.2. Modelling the bound state with the *PanDDA* method   

**Figure 2:**
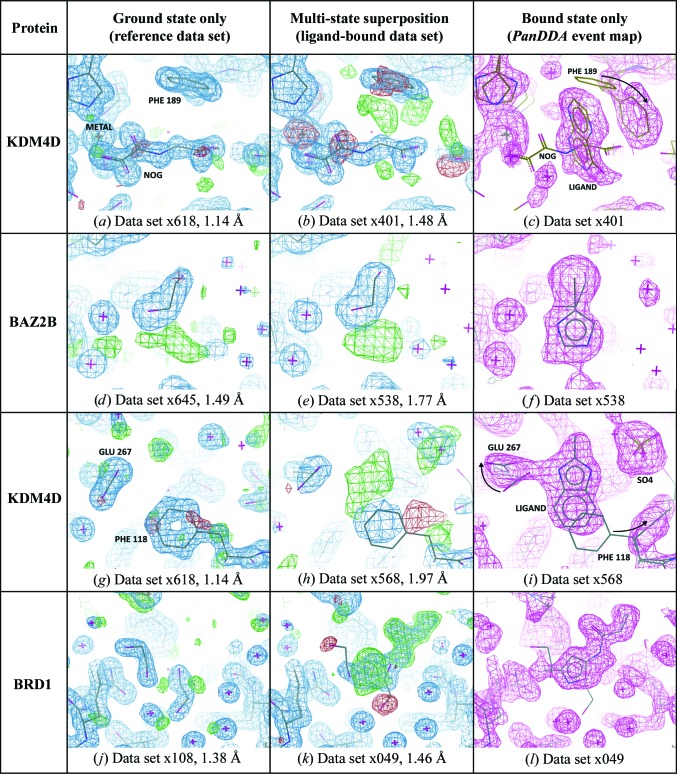
Determining the different crystal states requires different data sets. First two columns, 2*mF*_o_ − *DF*_c_ maps contoured at 1.5σ (blue) and *mF*_o_ − *DF*_c_ maps contoured at ±3σ (green/red). Last column, *PanDDA* event maps (blue) contoured at (*c*, *f*, *l*) 2σ or (*i*) 1σ. Resolutions are as indicated. First column, a reference data set provides the ground-state model of the crystal. Centre column, the ground-state refined into a ligand-bound data set leaves (generally uninterpretable) residual density for a superposed state. Last column, the *PanDDA* event map provides clear density for the ligand-bound model of the crystal (the superposed ground-state model is also shown for reference). (*a*, *b*, *c*) Example from §[Sec sec3.1]3.1. (*d*, *e*, *f*) Example from §[Sec sec3.2]3.2. (*g*, *h*, *i*) Example from §[Sec sec3.3]3.3. (*j*, *k*, *l*) Example from §[Sec sec3.4]3.4. In (*c*) and (*i*) arrows indicate side-chain changes from the ground-state to the bound-state model.

For the models presented in this work, the bound states are determined through use of the *PanDDA* method (Pearce *et al.*, 2016 ▸), where additional maps are available for the modelling of the ligand-bound conformation of the crystal. The *PanDDA* method compares a collection of multiple related crystallographic data sets, and identifies regions of individual data sets that are statistical outliers, *i.e.* regions where a bound state is present. The crystallographic superposition of bound and unbound states in the identified regions is then separated by subtracting a fraction of the ground-state electron density for the crystal, thereby revealing density for only the bound state of the crystal (for example the last column in Fig. 2 ▸) in a partial-difference map termed an ‘event map’.

Once a model for the bound state of the crystal has been constructed in the event maps, the *PanDDA* implementation automatically combines the bound-state model and the ground-state model to generate an ensemble model; where an accurate and complete ground-state model is used as the starting model for analysis with *PanDDA*, this automation greatly simplifies the modelling process and allows ensembles to be utilized with little additional effort. The assignment of the logical conformer IDs described in §[Sec sec2.1.1]2.1.1 is automatically performed during the merging process, and the *PanDDA* implementation further contains scripts to extract the individual states from the final ensemble. The ensembles thus generated were then refined using standard resolution-dependent refinement protocols; the occupancies of superposed states were constrained to sum to unity.

### 2.2. Comparison of different model types   

To establish whether the ensemble model does provide an improved description of the crystal, we prepared and compared three different types of model for each of the four data sets in §[Sec sec3]3: a bound-state-only model, an optimal ensemble model and a degraded-phase ensemble model (described below). A ground-state-only model was also refined for completeness (centre column in Fig. 2 ▸).

The bound-state-only model for refinement was obtained by removing the ground state from the ensemble and setting the occupancy of the bound state to 0.95 to trigger automated occupancy refinement in *phenix.refine* (Afonine *et al.*, 2012 ▸). The ground-state-only model was similarly generated by removing the bound state and setting the ground-state occupancy to 1.0, corresponding to the normal modelling case, where the occupancy of solvent molecules would not typically be refined.

To compare the effects of global phase degradation, degraded-phase ensemble models were produced for each of the examples in §[Sec sec3]3. The final high-quality ensemble model was distorted in regions distant from the ligand-binding site, thereby introducing a global phase error (Supporting Information §S1). Thus, the region around the binding site remains ‘optimally’ modelled, but the overall quality of the model has been neglected. Induced mean model phase difference relative to the full ensemble model is in the range 20–30° (as calculated by *CPHASEMATCH*; Winn *et al.*, 2011 ▸) and the *R*_free_ of the refined models is increased by around 10–15%.

All models were refined with *phenix.refine* v.1.9-1682 (Afonine *et al.*, 2012 ▸) against the relevant ligand-bound crystallographic data set, using the default parameters. Ligand occupancy was refined for all models; for the ensemble models, the occupancies of superposed states were constrained to sum to unity.

#### 2.2.1. Validation metrics for quantitative model comparison   

We compared the different modelling approaches using a variety of density-based and model-based validation metrics; these metrics and their optimal values are described in Table 1 ▸. Density metrics, all calculated by *EDSTATS* (Tickle, 2012 ▸), included the conventional real-space correlation coefficient (RSCC) and also the newer metrics of real-space *Z*-difference (RSZD) and real-space *Z*-observed (RSZO) scores (Tickle, 2012 ▸).

The RSCC measures the overall agreement between the model and the data; values of 0.7 or higher indicate a strong similarity between the model and the data. However, Tickle (2012 ▸) shows that the new metrics RSZD and RSZO can be used to ask more detailed questions about the model: RSZD measures the accuracy of the model through the analysis of difference density, highlighting modelling errors, and RSZO measures the precision of the density for the model, highlighting weak features.

RSZO is calculated by taking the average of the density over a residue and dividing by the noise in the map; since the amount of density for a residue is directly related to the occupancy of the residue, we divided RSZO by the occupancy of the residue to give a normalized value (RSZO/OCC) that could be used to compare models refined to different occupancies against the same data set.

We also calculate the *B*-factor ratio of the ligand to the surrounding protein residues (within 4 Å) as a measure of the consistency of the ligand model with its local environment, and the root-mean-squared deviation (r.m.s.d.) between the refined ligand and the high-quality ensemble ligand model coordinates as a measure of the (in)stability of the ligand model coordinates in refinement.

These five quality measures are displayed visually as radar plots for a single residue (for example Fig. 4), where the ‘better’ the metric value the closer it is to the centre of the plot. The axes of the radar plot in Fig. 4 are scaled such that the ‘best’ value is plotted at the centre of the plot and the ‘worst’ value is plotted at the extreme of the axis. For more information, see Supporting Information §S2.

## 3. Results   

We present four contrasting examples where the inclusion of a complementary solvent model leads to a better description of the crystal, and thereby to a higher quality ligand model. The ligands here were all identified and modelled using the *PanDDA* method (Pearce *et al.*, 2016 ▸). The model of the ligand was in each case derived from *PanDDA* event maps, and we investigate here only the effect that the inclusion/absence of the superposed solvent model has on the interpretation of the data. Models are generated and refined as described in §[Sec sec2.2]2.2. Validation metrics are calculated for only the ligand residue in each of the models. Crystallographic model parameters, including ligand validation scores, may be found in Supplementary Tables S1–S4. The chemical structures of the modelled compounds are shown in Supplementary Fig. S2.

### 3.1. Binding of a ligand at the same site as a bound substrate mimetic   

**Figure 3:**
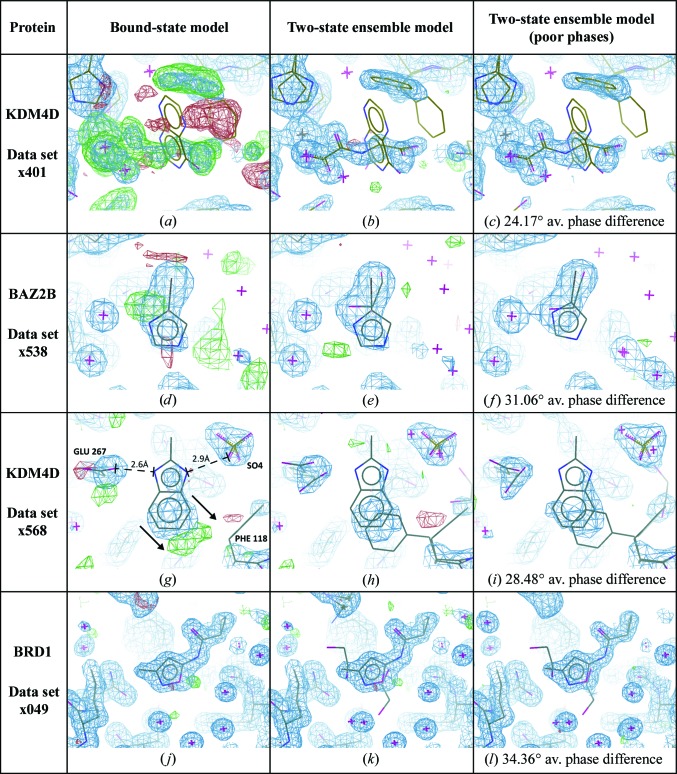
Ensemble models consistently have less residual difference density than ligand-only models. All images show 2*mF*_o_ − *DF*_c_ maps contoured at 1.5σ (blue) and *mF*_o_ − *DF*_c_ maps contoured at ±3σ (green/red). First column, refinement with the ligand model only. Centre column, refinement of the crystal as an ensemble of states. Last column, refinement of the crystal as an ensemble of states with a degraded protein model (phase difference as indicated relative to the ensemble model). (*a*, *d*, *g*) Modelling the ligand but removing the ground state leads to difference density for the absent state, and in (*d*) the ligand moves into density for the ground state (r.m.s.d. of 0.20 in Fig. 4 ▸ *b*). Arrows in (*g*) indicate the potential remodelling of the ligand by an overzealous modeller, as discussed in §[Sec sec3.3]3.3. (*j*) Removing the ground state for a high-occupancy ligand (refined value of 0.89) does not lead to discernible difference density. (*b*, *e*, *h*, *k*) Refinement of ensemble models explains all of the observed density, and ligands do not move from the fitted pose (confirmed by the validation plots in Fig. 4 ▸). (*c*, *f*, *i*, *l*) Refining with degraded phases leads to only minor visual differences in the model, except in (*f*) where the ligand moves significantly relative to the fitted pose. However, more striking is the disappearance of difference density, as expected, owing to the increase in the noise level of the map.

To demonstrate the process of modelling both states, we first present an example in which a weakly bound soaked ligand binds at the same site as a strongly bound substrate mimetic (*N*-oxalylglycine; NOG) and an ensemble is clearly necessary. The NOG molecule is tightly bound at high occupancy (∼90%) in the ground-state crystal form of human lysine-specific demethylase 4D (KDM4D), as shown in the reference data set (Fig. 2 ▸ *a*). However, in a ligand-bound data set a soaked ligand is present at the same site as the NOG, but in a perpendicular orientation, in only a small fraction of the crystal, as shown in the *PanDDA* event map (Fig. 2 ▸ *c*). Ligand binding is accompanied by a reordering of Phe189. Using the two maps (Figs. 2 ▸ *a* and 2 ▸ *c*), modelling of the two states can be performed separately, and the two models merged for refinement; when refined, the ensemble leads to a good model, with negligible amounts of difference density remaining (Fig. 3 ▸ *b*).

**Figure 4:**
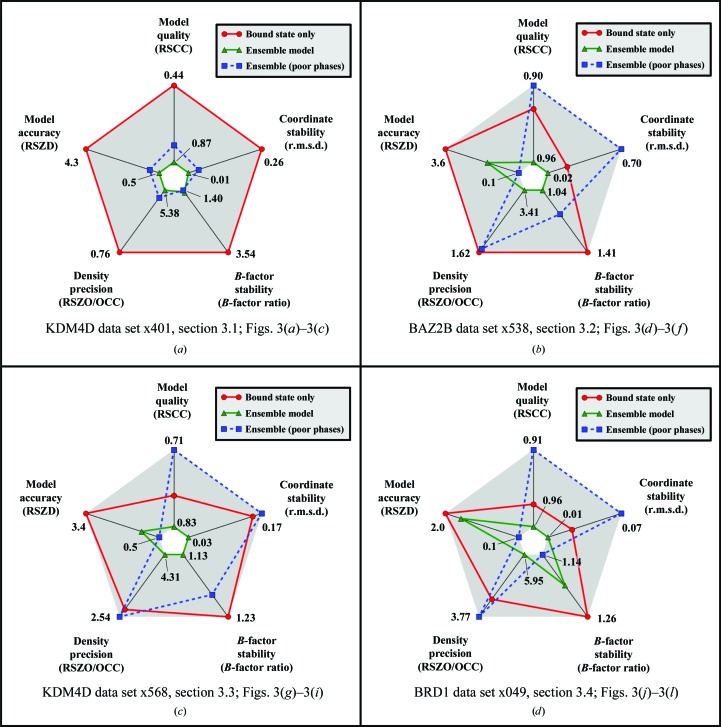
Validation plots for the different modelling approaches: axes are not absolute, but have been scaled relative to the minima and maxima of the plotted values, and only the minimum and maximum values are marked on the axes; for all model scores refer to Supporting Information §S1. (*a*) Plots for Figs. 3 ▸(*a*)–3 ▸(*c*). The plot confirms the visual inspection of the electron density; the ligand scores are improved across all metrics when refined as an ensemble relative to the ligand modelled alone. The absence of the superposed substrate model has a greater effect on the ligand model than the degradation of the protein model phases. (*b*) Plots for Figs. 3 ▸(*d*)–3 ▸(*f*). The ensemble model provides the best model for the ligand. The RSZD is decreased in the degraded-phase model for the reasons explained in the main text and is not related to an improved model. (*c*) Plots for Figs. 3 ▸(*g*)–3 ▸(*i*). Once more, the model statistics are improved with the addition of a superposed solvent model, with the caveat that the lower RSZD for degraded phases is not indicative of an improved model. (*d*) Plots for Figs. 3 ▸(*j*)–3 ▸(*l*). The inclusion of the solvent model still increases the quality of the model compared with when it is omitted, albeit marginally. The degraded phase model has lower *B*-factor ratios than either of the other two models owing to a decrease in the *B* factors of the ligand and a corresponding drop in occupancy.

Although not interpretable, residual difference density can still be seen for the bound ligand when the ground-state model is refined alone (Fig. 2 ▸ *b*). As expected, refinement of the ligand without the superposed NOG results in a poor-quality model (Fig. 3 ▸ *a*) because a large fraction of the crystal is locally unrepresented; refinement of the ensemble results in a better model for the ligand (Fig. 3 ▸ *b*), scoring well across all five metrics. On the radar validation plot (Fig. 4 ▸ *a*) this is shown as the ensemble-model line (green, triangles) being entirely contained within the ligand-only line (red, circles): the closer the line is to the centre of the plot, the better the model. Sensible refinement of the ligand requires the superposed ground-state conformation to be present.

The degraded-phase ensemble model (Fig. 3 ▸ *c*) has a 24.17° average phase difference from the high-quality ensemble model, increasing the *R*_free_ from 17 to 29%. However, the model of the ligand is not significantly degraded (blue line, squares; Fig. 4 ▸ *a*), and still scores well on all five model validation metrics, although worse than the ensemble model with high-quality phases. In this case, the omission of the local model has a qualitatively larger impact than the quality of the global phases.

### 3.2. Binding of a ligand in place of a solvent molecule   

In a soaked crystal of human bromodomain adjacent to zinc-finger domain 2B (BAZ2B), an ethylene glycol is bound in a semi-ordered fashion, with a superposed ligand, to the asparagine in the binding site. The solvent ground-state model derived from a reference data set is not optimal, and some difference density remains even when a soaked (non-ethylene glycol) ligand is not present (Fig. 2 ▸ *d*). Refinement with the ground-state model in the ligand-bound data set does not lead to significant additional difference density, as the refined solvent model masks the presence of the Br atom of the ligand (Fig. 2 ▸ *e*).

The *PanDDA* event map, however, shows clear evidence for the ligand (Fig. 2 ▸ *f*); the positioning of the bromine can also be confirmed by an anomalous difference map (Supporting Information; Pearce *et al.*, 2016 ▸). Refinement with only the bound state causes the ligand atoms to be pulled into the density for the ethylene glycol, and difference density remains (Fig. 3 ▸ *d*). Refinement of the ensemble leads to a good model (Fig. 3 ▸ *e*), with all density well explained and no movement of the ligand from the fitted pose. Refinement of the degraded-phase model (Fig. 3 ▸ *f*) also causes the ligand to move relative to the fitted position.

In this case, the absence of the superposed model and the quality of the model phases are both as important for the quality of the final ligand model, as reflected by the validation metrics (Fig. 4 ▸ *b*). It should be noted that the residual density from the ground state (Fig. 2 ▸ *d*), which is not accounted for by the ground-state model, will have caused the ligand occupancy to be overestimated in refinement; this exemplifies issues with our inability to perfectly model the ground state.

It is noteworthy that the RSCC of the ligand in all models is greater than 0.9, showing that whilst a large RSCC is necessary for a good model, it is not sufficient to determine the quality of the model: it does not account for the presence of difference density. As discussed in §[Sec sec4.3]4.3, the RSZD of 0.1 for the degraded-phase ligand model, which would normally indicate a very good model, is affected by noise in the maps from the degraded phases; the RSZD is very sensitive to the global accuracy of the model and the corresponding quality of the phases. Multiple validation metrics, as well as a near-complete model, are needed to validate weak features.

### 3.3. A binding ligand overlaps with alternate conformations of a side chain   

Another ligand in a KDM4D data set binds along with a sulfate to a putative allosteric site. Refinement with the ground-state conformation leaves residual unmodelled difference density (Figs. 2 ▸ *g* and 2 ▸ *h*). The pose and identity of the ligand is clearly revealed in the *PanDDA* event map (Fig. 2 ▸ *i*), revealing the reordering of two side chains and that the ligand is superposed on the ground-state conformation of the phenylalanine (Phe118).

Upon inspection of the refined ensemble model (Fig. 3 ▸ *h*), it was suggested to the authors by another experienced crystallographer that the ground-state conformation should be deleted and the ligand-bound state refined as the sole conformation. This anecdote supports our observation that the pervading convention, to generate only a single conformation of the crystal wherever possible, dominates even in the face of clear evidence that multiple states are present. The density in the area of overlap between the ligand and the phenylalanine is significantly stronger than over the rest of either residue, and difference density is present when either state is refined separately (Figs. 2 ▸ *h* and 3 ▸ *g*). The residual density from the bound-state-only model (Fig. 3 ▸ *g*) might further tempt a crystallographer to move the ligand model down and right by ∼1 Å (as indicated by arrows in Fig. 3 ▸ *g*), although this causes clashes with the C^β^ atom of Phe118 and adversely affects the interactions that the ligand makes with Glu267 and a bound sulfate (Fig. 3 ▸ *g*). All evidence points towards the presence of multiple states in the data, and therefore these multiple states should be present in the model.

The phase degradation in Fig. 3 ▸(*i*) (mean phase difference to the ensemble model of 28.48°) degrades the ligand model RSZO and the *B*-factor ratio to a similar level as the omission of the ground-state model and significantly degrades the RSCC (Fig. 4 ▸ *c*). Again, we observe an expected decrease in the RSZD with the decrease in phase quality. In summary, the high-quality ensemble model provides the best interpretation of the experimental data.

### 3.4. Traces of the ground state remain, even for a high-occupancy ligand   

One ligand screened against the bromodomain of BRD1 binds strongly in the principal binding site (Figs. 2 ▸ *j* and 2 ▸ *k*), with a refined occupancy of 84–89% (multi-state and bound-state-only refined occupancies, respectively). In the reverse case to §[Sec sec3.1]3.1, the ligand occupancy is much higher than the ground-state occupancy, and this ligand would conventionally be modelled at unitary occupancy, with the resulting error absorbed by the *B* factors.

Once more, inclusion of the ground-state solvent improves the model quality, although in this case only marginally (Figs. 3 ▸ *j*, 3 ▸ *k* and 4 ▸ *d*). Even with this strong binder, visual traces of the ground-state model remain, although certainly not appropriate for modelling; contouring the 2*mF*_o_ − *DF*_c_ map to 0σ indicates weak evidence that the ground-state solvent is still present, because it shows density where the model suggests that it should be (Supplementary Fig. S3). Furthermore, there is no difference density after refinement to propose the removal of the ground-state model (Fig. 3 ▸ *k*).

Phase degradation degrades the RSCC, RMSD and the RSZO more than the absence of the solvent model, with a decrease in the RSZD as previously. Here, the *B*-factor ratio is seen to be lower for the phase-degraded model than for the other models owing to a decrease in the *B* factors of the ligand by two and a corresponding decrease in the occupancy to 0.77; this behaviour demonstrates the ambiguity that can be observed in simultaneous refinement of *B* factors and occupancies.

## 4. Discussion   

The examples presented here provide consistent evidence for ground-state molecules co-existing with ligand-bound molecules in crystals across a range of non-unitary occupancies. Moreover, the inclusion of a superposed ground-state model, obtained from a reference data set, improves the quality of the obtained ligand models in all cases. In the case of some weak ligands, the ground state model is crucial for the refinement of the protein–ligand complex (§[Sec sec3.1]3.1); in other cases it acts simply to remove ‘extraneous’ difference density that could be interpreted by an overzealous modeller as being caused by a ligand in multiple conformations (§[Sec sec3.1]3.2). The modelling approach can affect the interpretation of intermolecular interactions (§[Sec sec3.3]3.3), and in the case of high occupancy a superposed ground state can still marginally improve the ligand model, alongside providing a more complete model of the crystal (§[Sec sec3.4]3.4).

Our experience to date suggests that at the resolutions reported here (better than 2 Å) it is invalid to remove the ground state from the ensemble model if the occupancy of the ground-state conformer refines to values above 10%. The cutoff might be less stringent at lower resolutions, but how to determine it, and how it relates to overfitting, is the subject of ongoing work.

In general, however, we conclude that, contrary to the current convention, one must assume the ground state to be present in the ligand-bound crystal until it is proven absent. This invokes the thinking articulated two decades ago, when rigorous validation became established best-practice principle (Kleywegt & Jones, 1998 ▸), that strong physical assumptions and restraints should apply by default, and can only be removed when justified by strong counter-evidence. Thus, unless it is clear that the ground-state model is problematic (*e.g.* unstable refinement), it should be retained.

At the same time, the usual concerns around over-parametrizing the crystallographic model apply, and become more acute at lower resolutions. While the pitfalls remain to be fully characterized and fall outside the scope of this work, the main problems we foresee are the numerical stability of the *B*-factor and occupancy values, and the reliability of the validation metrics. The former would be significantly stabil­ized by restraining the ground-state atoms to a reference data set (see, for example, Smart *et al.*, 2012 ▸; Nicholls *et al.*, 2012 ▸; Headd *et al.*, 2012 ▸), and this should almost certainly be considered best practice. However, it is currently technically nontrivial to configure and we have therefore not yet assessed this in practice; the details of implementation are a work in progress.

There is some urgency in resolving the details of best practice: the increase of crystallographic fragment-screening experiments amongst academic groups is set to produce a sharp increase in structures in the PDB containing sub-unitary occupancy binders (see, for example, Schiebel *et al.*, 2016 ▸), which we show benefit most from this superposition modelling.

### 4.1. Practical considerations   

Generating an atomic model of the ground state requires an independent ground-state data set. This is usually available or easy to obtain where bound-state crystals are generated by soaking; these experiments are also most likely to benefit from explicit ground-state modelling, since soaking seldom guarantees full-occupancy binding. The same is true for ligands introduced by co-crystallization that produce a crystal form identical to crystallization without ligand: here, the bound state is more likely to be near full occupancy, but there is no reason to assume this in general.

On the face of it, the ensemble strategy seems least relevant where co-crystallization with ligand yields new crystal forms that are (or appear to be) contingent on conformational changes induced by the ligand. Here, one is led to assume that the bound state is invariably at full occupancy, and this is probably commonly true. Nevertheless, in the general case, the assumption does not hold up to scrutiny of the physics of crystallization: as long as there is no energetic penalty for the unbound state to sample the same conformation induced by the ligand, protein molecules in the unbound state should be able to join the growing crystal lattice as ground-state molecules, leading again to sub-unitary occupancy. The symptoms of such behaviour would be unexplained difference density in the binding site, and in these cases ground-state crystals should be readily obtainable, without damaging the lattice, by leaching ligand out of the crystal by suitable back-soaking protocols, or alternatively by seeding.

Computationally, uptake of the approach requires the implementation of tools for the trivial generation of ensembles from multiple single-state models; the *PanDDA* implementation goes some way towards achieving this, although more work is required. Performed correctly, the addition of a superposed ground-state model allows no further freedom for a crystallographer to overinterpret the ligand-bound data-set density, as the ground-state model is solely determined in an independent reference data set. The only risk of overfitting remains in refinement, and here the protocols will further improve over time, in particular the application of external restraints.

An alternative approach to accounting for the ground state would be to model this fraction as bulk solvent. We are not aware of this being used in any current refinement program, and it is unlikely to be straightforward, considering that the modelling of bulk solvent remains an open question, even outside of binding sites (Weichenberger *et al.*, 2015 ▸). Nevertheless, the importance of explicitly considering bulk solvent in the binding site is illustrated by its effectiveness in the related question of improving OMIT density (Vonrhein & Bricogne, 2005 ▸; Liebschner *et al.*, 2017 ▸).

Valid ensemble-construction protocols can lead to complicated models and refinement constraints that are currently not supported by the refinement programs that we worked with [*REFMAC* (Murshudov *et al.*, 2011 ▸) and *phenix.refine* (Afonine *et al.*, 2012 ▸)]. In some cases that are not shown here, we have found that constraining the occupancies of multiple-conformer models in refinement permitted occupancies for amino acids that summed to greater than unity. Further work is therefore required to automatically generate occupancy and structural restraints that allow complex ensemble refinement in the general modelling case without permitting unphysical atomic models; procedural generation of such parameterization files will be critical to the uptake of this approach, and will be deployed within *PanDDA* as they are developed.

In addition, the limitations of alternate conformers can quickly manifest themselves when merging models that contain multiple conformations in the bound-state and ground-state models. The alternate conformer-modelling formalism does not support branching of conformations, where, for example, an alternate conformation of the backbone can have two side-chain conformers. In regions where either the ground state or the bound state have multiple conformations, it may be necessary to duplicate single-conformer residues to generate a contiguous ensemble model. The addition of redundant atoms in such cases increases the number of model parameters, and thus increases the potential for overfitting during refinement; the use of tight external restraints on duplicated atoms during refinement are necessary to remove these additional free parameters. Robust and general methods to perform the merging of multiple states, and those to generate the required refinement restraints algorithmically, to reduce the number of model parameters, remain the subject of ongoing work.

### 4.2. Validation   

Our examples highlight that the RSCC alone is not enough to assess the quality of a ligand model, and emphasize the need for the additional metrics introduced here. Model correctness is also reflected in RSZD and RSZO, as long as the phases are near convergence, while the stability of refinement of *B* factors and model coordinates are captured by the *B*-factor ratio and r.m.s.d., respectively. Finally, the stability of the *B*-factor and occupancy refinement is assessed by the combination of a normalized RSZO and *B*-factor ratio: an imbalance indicates over-parametrization or inadequate restraints.

In practical terms, this means the following: model *completeness* of the overall model is reflected in the RSCC and RSZD; however, they do not inform on its quality, except where poor values are obtained, as they can always be expected to improve as more model parameters are added. Model *quality* is instead chiefly indicated by the *B*-factor ratio, whilst the *parametrization* is assessed by the interplay between the normalized RSZO and *B*-factor ratio. The r.m.s.d. provides a measure of model coordinate *stability*. It is thus the combination of all five metrics that *validates* the model in a wide range of refinement scenarios.

**Figure 5:**
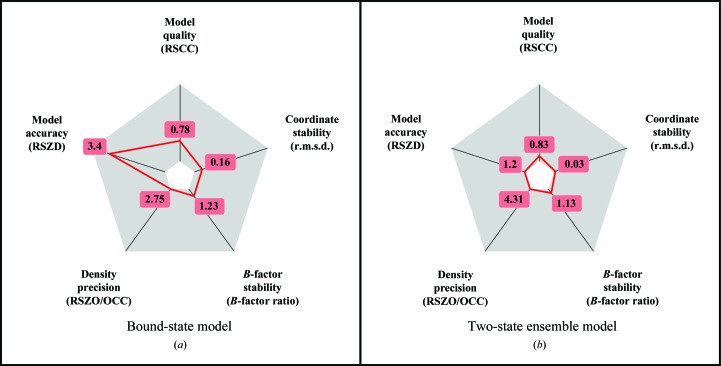
Radar plots clearly display the suspect features of a ligand and indicate when the validation scores deviate from ideal values. Validation plots for the ligand in §[Sec sec3.3]3.3 are shown in (*a*) for the ligand-only model and in (*b*) for the ligand when refined as an ensemble (scores are for the ligand residue only). Limits and thresholds for the validation plots are detailed in Supplementary Table S5. The ligand-only model shows that unmodelled features are present with a large RSZD. The ensemble model (with high-quality phases) scores well on all metrics, and remains close to the centre of the plot.

The radar plots used here present the validation metrics clearly, and may be a useful tool for the validation of ligands in general. In this manuscript, we have used the validation plots to compare multiple models, and to this end the plot axes were rescaled to cover the range of the data. However, a more general use of the radar plot is to show when the ligand scores depart from ideal values (the proposed ranges for the metrics are shown in Supporting Information §S2); an example is shown in Fig. 5 ▸ for the ligand described in §[Sec sec3.3]3.3.

Like many validation metrics, the ones that we propose are weakened as resolution decreases, since they rely on such things as the numerical stability of the *B* factor and occupancy refinement. Here, the ability to restrain *B* factors to a reference structure is likely to be important, and will be assessed as these features become available in refinement programs.

### 4.3. Model completeness and accuracy: local *versus* global   

Long-established crystallographic teaching holds that the optimal crystallographic model can only be obtained if phases are maximally accurate, because only then will the maps show all resolvable subtleties necessary for building the best model. This approach certainly extends to ligand-binding studies, and best practice is to correct all detectable model errors, even if minor and structurally remote, before attempting to model the ligand and associated changes. Recent reports demonstrate systematically that this is indeed effective at improving the interpretation of weak difference density (Schiebel *et al.*, 2016 ▸), although not actually proving the assertion, not least because the authors did not set out to do so.

Our work suggests that near convergence things are more nuanced: in particular, as phases improve they eventually cease to be the dominant source of error in the model. Here we identify a different source of error, namely incomplete modelling of the superposition of states that can be defended from first principles.

We now submit that this ‘superposition error’ dominates local map quality as phases approach convergence. In at least one example (§[Sec sec3.1]3.1), the superposition error is very large indeed and more significant than even a very large global phase error, as shown in Fig. 4 ▸(*a*), where all validation metrics, even the phase-sensitive one (RSZD), improve from the degraded phase to the ensemble models. More typically, the crossover point (the phase error below which the superposition error dominates) will tend to lie far closer to phase convergence; this is evidently the case for the other three examples, and more work is needed to understand this behaviour in general.

What will always require good (enough) phases are the phase-sensitive metrics (RSZD and RSZO), which are only reliable and informative near convergence: their derivation assumes accurate phases because they are inversely proportional to the noise in the overall electron-density map (Tickle, 2012 ▸), which is increased by poorer phases. In general, density-based model validation requires high-quality data near the end of the refinement process; this is where the ensemble-based approach may best be validated.

Knowing the crossover point has a practical use: once the model has improved beyond it, it becomes unproductive to spend more time improving distant parts of the model, and its is more important instead to model the superposition at the binding site of interest. Our experience to date indicates that for straightforward ligand-binding studies a single round of refinement starting from a good reference (ground-state) model will generally suffice to bring a model to a state that, subjectively gauged, behaves as we would expect past the crossover point. The implication is that in these kinds of ‘molecular substitution’ studies, the main or even only thing required to complete the model to sufficient accuracy is to generate the ensemble and assess its refinement. Certainly this topic requires much more systematic study, however this lies outside the scope of this report.

## 5. Data availability   

All crystallographic data for the various versions of each model have been uploaded to Zenodo (https://doi.org/10.5281/zenodo.228000). The KDM4D structures are labelled as JMJD2D for consistency with the original *PanDDA* manuscript. Interactive HTML summaries for all of the fragment-screening data sets can also be found at https://zenodo.org/record/290220/ (for JMJD2D), https://zenodo.org/record/290199/ (for BAZ2B) and https://zenodo.org/record/290217/ (for BRD1).

## 6. Related literature   

The following references are cited in the Supporting Information for this article: Lang *et al.* (2014 ▸).

## Supplementary Material

Supporting Information.. DOI: 10.1107/S2059798317003412/ba5259sup1.pdf

Crystallographic data for the different versions of each model. URL: https://doi.org/10.5281/zenodo.228000

PanDDA analysis of JMJD2D screened against Zenobia Fragment Library. URL: https://doi.org/10.5281/zenodo.290220

PanDDA analysis of BAZ2B screened against Zenobia Fragment Library. URL: https://doi.org/10.5281/zenodo.290199

PanDDA analysis of BRD1 screened against 3D-Fragment-Consortium Fragment Library. URL: https://doi.org/10.5281/zenodo.290217

## Figures and Tables

**Table 1:** Electron-density and model metrics used for the validation of crystallographic models The combination of five metrics highlights a variety of features of models and together they allow a comprehensive description of the atomic model of a residue. RSCC ensures good overall similarity of the model to the density. RSZD measures the difference density over the model, highlighting errors or the presence of currently unmodelled or over-modelled atoms. RSZO indicates density strength, and the normalization by occupancy can indicate errors in the occupancy of a model or a misplaced or absent model. The *B*-factor ratio highlights errors in the *B* factors of a residue, as these should be consistent with its surroundings: physically, there cannot be step changes in mobility of atoms in a crystal. The r.m.s.d. measures the movement of residues in refinement; a numerically unstable residue may be indicative of error in the model. All density metrics were calculated using *EDSTATS* (Tickle, 2012 ▸).

Metric	Description	Preferred values
RSCC	Correlation between model and observed electron density	>0.7
RSZD	Statistical measure of difference density in region of model	<3
RSZO/OCC	Strength of density over model, normalized for occupancy	>2
*B*-factor ratio	*B*-factor ratio of residue atoms and side-chain atoms within 4 Å	∼1
R.m.s.d.	Root-mean-squared deviation of the atomic coordinates (Å)	<1
