# Supplementary material for: Proper modelling of ligand binding requires an ensemble of bound and unbound states
Source: Acta Crystallogr D Struct Biol. 2017 Mar 6;73(Pt 3):256–66. doi: 10.1107/S2059798317003412 (PMC5349438; doi:10.1107/S2059798317003412)
Supplement: Supplementary file 1 [file d-73-00256-sup1.pdf]

STRUCTURAL BIOLOGY

Volume 73 (2017)

Supporting information for article:

Proper modelling of ligand binding requires an ensemble of bound and unbound states

Nicholas M. Pearce, Tobias Krojer and Frank von Delft

Supporting information

S1. Crystallographic Information for Examples

The crystallographic parameters for each of the models used in the examples are listed in Table S1-Table S4. All models are refined with phenix.refine (Afonine *et al.*, 2012) using the standard settings. All phase differences are calculated with cphasematch (Winn *et al.*, 2011) from the model phases as output by phenix.refine, relative to the ensemble-model phases. Occupancy refinement was performed on all models, except for the ground-state-only model. When multiple conformations were modelled, the occupancies were constrained to sum to unity.

S1.1. Generation of the degraded-phase models

Degraded-phase models were generated using the real-space refinement tool in Coot (Emsley *et al.*, 2010); residues on the surface of the protein distant to the binding site were manually dragged out of position, thereby also pulling the local section of polypeptide backbone out of position. When released, the structure relaxes under the real-space refinement restraints in coot. This process was repeated several times at various points on the protein surface. The result of this is a model of the protein where the binding site is the same model as the "optimally-refined" structure of the protein, but where significant regions elsewhere on the protein are poorly modelled. The RMSDs between the refined ensemble models and the corresponding degraded-phase models can be seen in Table S1-Table S4, and typically fall in the region of 2.35-3.25Å all-atom RMSD.

S2. Validation Radar Plots

Standard validation plots are generated by recording the density scores radially on the graph axes and connecting these points with lines. For the comparative plots (Figure 4), the axes are re-scaled such that the limits are the minimum and maximum of the metric scores. For normal validation plots (Figure 5), the limits of each of the scores are shown in Table S5. These plots can be generated using the giant.score_model script distributed as part of the giant package within the panddas package, available as part of CCP4 (Winn *et al.*, 2011).

Table S1 Crystallographic parameters and ligand model scores for the models of KDM4D dataset x401 (Section 3.1). All-atom RMSD of the degraded-phase model and the ensemble model after refinement was 2.351Å. "Bound state" refers to the state where the "ligand of interest" is bound; the ground state may also contain bound ligands (e.g. solvent or buffer molecules).

Model	Mean Phase Diff. (°)	R-work/ R-free	Occ.	RSCC	RSZD	RSZO/ OCC	B-factor Ratio	RMSD (Å)
Ground State Only (Figure 2b)	2.92	0.129 / 0.171	-	n/a	n/a	n/a	n/a	n/a
Bound State Only (Figure 3a)	9.97	0.147 / 0.195	0.79	0.44	4.3	0.76	3.54	0.26
Ensemble Model (Figure 3b)	-	0.127 / 0.171	0.26	0.87	0.5	5.38	1.47	0.01
Degraded Ensemble (Figure 3c)	24.17	0.241 / 0.290	0.27	0.77	1.0	4.81	1.40	0.05

Abbreviations: Occ, ligand occupancy; RSCC, real-space correlation coefficient; RSZD, real-space Z-difference score; RSZO, real-space Z-observed score; RMSD, coordinate root-mean-squared difference.

Table S2 Crystallographic parameters and ligand model scores for the models of BAZ2B dataset x538 (Section 3.2). All-atom RMSD of the degraded-phase and ensemble model after refinement was 2.625Å. Conventions and abbreviations are as in Table S1.

Model	Mean Phase Diff. (°)	R-work/ R-free	Occ.	RSCC	RSZD	RSZO/ OCC	B-factor Ratio	RMSD (Å)
Ground State Only (Figure 2e)	2.95	0.183 / 0.217	-	n/a	n/a	n/a	n/a	n/a
Bound State Only (Figure 3d)	4.15	0.184 / 0.215	0.68	0.92	3.6	1.62	1.41	0.20
Ensemble Model (Figure 3e)	-	0.182 / 0.216	0.41	0.96	1.6	3.41	1.04	0.02
Degraded Ensemble (Figure 3f)	31.06	0.311 / 0.363	0.29	0.90	0.1	1.72	1.18	0.70

Table S3 Crystallographic parameters and ligand model scores for the models of KDM4D dataset x568 (Section 3.3). All-atom RMSD of the degraded-phase model and the ensemble model after refinement was 2.518Å. Conventions and abbreviations are as in Table S1.

Model	Mean Phase Diff. (°)	R-work/ R-free	Occ.	RSCC	RSZD	RSZO/ OCC	B-factor Ratio	RMSD (Å)
Ground State Only (Figure 2h)	3.86	0.157 / 0.219	-	n/a	n/a	n/a	n/a	n/a
Bound State Only (Figure 3g)	4.38	0.164 / 0.222	0.80	0.78	3.40	2.75	1.23	0.16
Ensemble Model (Figure 3h)	-	0.159 / 0.220	0.51	0.83	1.20	4.31	1.13	0.03
Degraded Ensemble (Figure 3i)	28.48	0.274 / 0.332	0.59	0.71	0.50	2.54	1.19	0.17

Table S4 Crystallographic parameters and ligand model scores for the models of BRD1 dataset x049 (Section 3.4). All-atom RMSD of the degraded-phase model and the ensemble model after refinement was 3.246Å. Conventions and abbreviations are as in Table S1.

Model	Mean Phase Diff. (°)	R-work/ R-free	Occ.	RSCC	RSZD	RSZO/ OCC	B-factor Ratio	RMSD (Å)
Ground State Only (Figure 2k)	4.22	0.186 / 0.216	-	n/a	n/a	n/a	n/a	n/a
Bound State Only (Figure 3j)	2.20	0.183 / 0.213	0.89	0.95	2.00	4.38	1.26	0.03
Ensemble Model (Figure 3k)	-	0.182 / 0.212	0.84	0.96	1.60	5.95	1.20	0.01
Degraded Ensemble (Figure 3l)	34.46	0.341 / 0.380	0.77	0.91	0.10	3.77	1.14	0.07

Table S5 Radar plot axes limits. The limits and length scales for the radial axes are defined here. The inner limit defines the value at which the plotted line will begin to move away from the centre of the plot. The outer limit defines the values at which the plotted line will reach the end of the radial axis, and be plotted outside the graph area. If a metric is inverted, large values will be plotted closer to the centre of the radar plot, and smaller values will be plotted further from the centre.

Metric	Inner Limit	Outer Limit	Inverted
RSCC	0.85	0.6	Yes
RSZD	1.5	4	No
RSZO/OCC	2	0	Yes
B-factor Ratio	1	3	No
RMSD (Å)	0	1.5	No

Abbreviations: Occ, residue occupancy; RSCC, real-space correlation coefficient; RSZD, real-space Z-difference score; RSZO, real-space Z-observed score; RMSD, coordinate root-mean-squared difference.

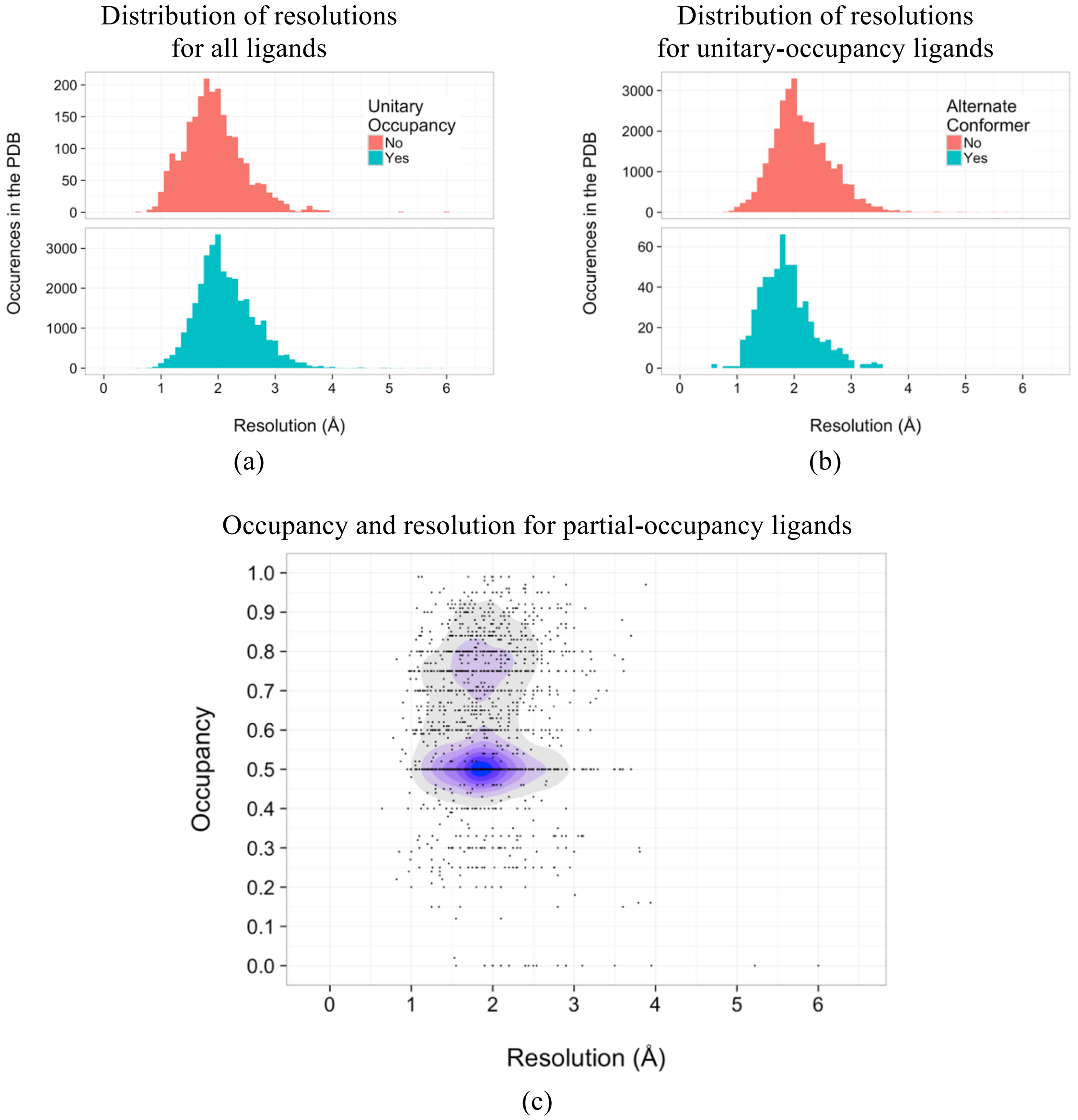

Figure S1 **Resolutions of ligand-bound structures are unrelated to incidence of non-unitary occupancy.** Dataset of ligands used is as in Figure 1; plots are truncated at 6.5Å. (a) Distribution of structure resolutions for all ligands, coloured by whether the ligand is modelled at unitary occupancy (unitary occupancy includes ligands modelled in multiple conformations that sum to unitary occupancy). The distribution peaks around 2Å, and 99.6% of structures have a resolution below 4Å. (b) Distribution of structure resolutions for unitary-occupancy ligands, coloured by whether the ligand is labelled with an alternate conformer. There is an increase in the utilisation of alternate ligand conformations at high resolution, as expected. (c) Contour plot of structure resolution and occupancy of the ligand for non-unitary occupancy ligands, bluer regions indicating higher densities of data: there is no strong relationship between the occupancy of sub-unitary occupancy ligands and the resolution of the structure. A scatter plot of the data is overlaid on the contour plot, but is not informative as the data are heavily overplotted at specific, neat fractions of occupancy (0.33, 0.5, 0.6, 0.75, etc.), which strongly suggests that those occupancies were manually selected rather than refined. (Ligands on rotational symmetry axes will have fractional symmetries, but will be rare in this dataset, given how the structures were filtered.)

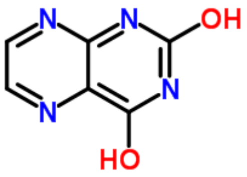

(a) c1cnc2c(c(nc(n2)O)O)n1

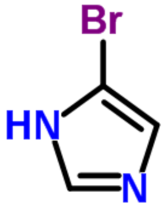

(b) c1c(nc[nH]1)[Br]

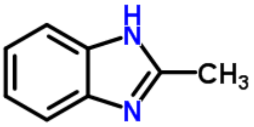

(c) Cc1nc2ccccc2[nH]1

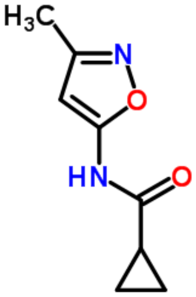

(d) Cc1cc(NC(C2CC2)=O)on1

Figure S2 Chemical structures and smiles strings of ligands. (a) Ligand from section 3.1. (b) Ligand from section 3.2. (c) Ligand from section 3.3. (d) Ligand from section 3.4.

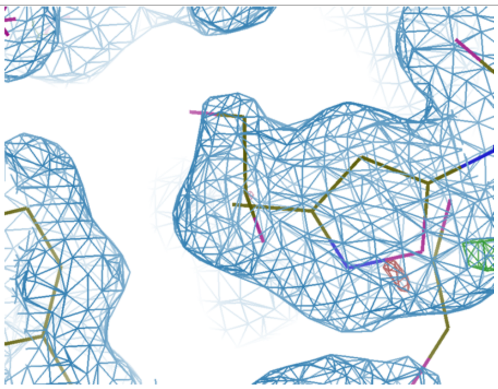

Figure S3 Weak density for the ground-state model is visible in refined maps even for a high-occupancy ligand. Same maps and model as in Figure 3k, but with $2mF_o$-DF_c map (blue) contoured at a very low level; the mF_o-DF_c map (green/red) is contoured at $\pm3\sigma$ as normal. The good contrast in the maps indicates they are contoured at an informative level, allowing the density around the ethylene glycol molecule to be taken as evidence of its presence. Numerically, the contour level is 0.0σ, but since maps were not placed on an absolute scale, that contour does not represent absence of electrons, nor does it represent a statistical level of significance (Lang *et al.*, 2014).

Supplementary References

Afonine, P. V., Grosse-Kunstleve, R. W., Echols, N., Headd, J. J., Moriarty, N. W., Mustyakimov, M., Terwilliger, T. C., Urzhumtsev, A., Zwart, P. H. & Adams, P. D. (2012). *Acta Crystallogr. Sect. D Biol. Crystallogr.* **68**, 352–367.

Emsley, P., Lohkamp, B., Scott, W. G. & Cowtan, K. (2010). *Acta Crystallogr. Sect. D Biol. Crystallogr.* **66**, 486–501.

Lang, P. T., Holton, J. M., Fraser, J. S. & Alber, T. (2014). *Proc. Natl. Acad. Sci.* **111**, 237–242.

Winn, M. D., Ballard, C. C., Cowtan, K. D., Dodson, E. J., Emsley, P., Evans, P. R., Keegan, R. M., Krissinel, E. B., Leslie, A. G. W., McCoy, A., McNicholas, S. J., Murshudov, G. N., Pannu, N. S., Potterton, E. a., Powell, H. R., Read, R. J., Vagin, A. & Wilson, K. S. (2011). *Acta Crystallogr. Sect. D Biol. Crystallogr.* **67**, 235–242.
